# Crystal structure of Cs_2_[Th(NO_3_)_6_]

**DOI:** 10.1107/S1600536814015876

**Published:** 2014-07-19

**Authors:** Patrick Woidy, Florian Kraus

**Affiliations:** a AG Fluorchemie, Department of Chemistry, Technische Universität München, Lichtenbergstrasse 4, 85747 Garching, Germany

**Keywords:** thorium, hexa­nitratothorate, crystal structure

## Abstract

Dicaesium hexa­nitratothorate(IV), Cs_2_[Th(NO_3_)_6_], was synthesized in the form of colourless crystals by reaction of thorium nitrate and caesium nitrate in aqueous solution. The Th atom is located on an inversion centre and is coordinated by six chelating nitrate anions. The resulting ThO_12_ coordination polyhedron is best described as a slightly distorted icosa­hedron. The Cs atom also has a coordination number of 12, but its coordination polyhedron is considerably more distorted. The crystal packing can be derived from an hexa­gonal dense packing (hcp) of idealized spherical CsO_12_ and ThO_12_ units. The CsO_12_ units form a distorted hcp arrangement and half of the octa­hedral sites are occupied by the ThO_12_ units.

## Abstract 2

The crystal structure of Cs_2_[Th(NO_3_)_6_] can be derived from a dense packing of idealized CsO_12_ and ThO_12_ units. The CsO_12_ units form a distorted hcp arrangement with half of the octa­hedral sites occupied by ThO_12_ units.

## Chemical context   

Nitrato complexes of the actinoids (Ryan, 1961 ▶; Strnad & Kohler, 1989 ▶) play an important role in the production of nuclear fuel as well as in its reprocessing. Moreover, multinary thorium nitrate compounds are of potential inter­est as anhydrous starting materials for further chemical conversion.

## Structural commentary   

**Figure 1:**
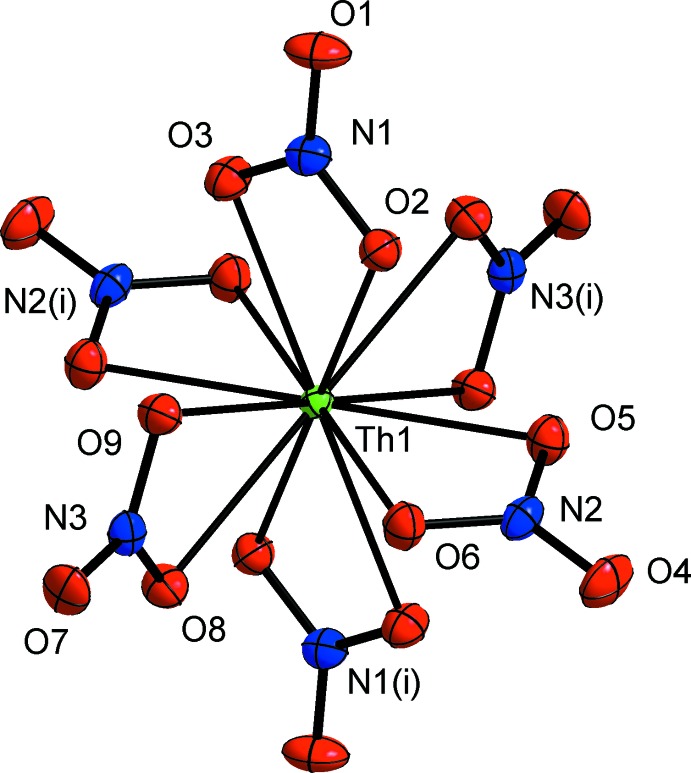
The [Th(NO_3_)_6_]^2−^-anion of the title compound. Displacement ellipsoids are drawn at the 70% probability level. Labelling for symmetry-equivalent oxygen atoms is omitted for clarity. [Symmetry code: (i) −*x*, −*y* + 1, −*z*.]

The thorium atom, Th1, occupies Wyckoff position 2*c* and has site symmetry 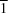. It is coordinated by six chelating nitrate anions in general positions. The resulting ThO_12_ polyhedron can be best described as a slightly distorted icosa­hedron. The [Th(NO_3_)_6_]^2−^-anion is shown in Fig. 1 ▶. Its Th—O distances are in a rather narrow range from 2.541 (2) to 2.581 (2) Å and compare quite well with Th—O distances of other reported thorium nitrate structures. In Th(NO_3_)_4_(H_2_O)_4_, they range from 2.54 (1) to 2.61 (1) Å (Charpin *et al.*, 1987 ▶), in Th(NO_3_)_4_(H_2_O)_5_ from 2.50 (1) to 2.62 (1) Å (Ueki *et al.*, 1966 ▶; Taylor *et al.*, 1966 ▶), and in the cubic structure of K_2_[Th(NO_3_)_6_] Th—O distances ranging from 2.535 (2) to 2.581 (2) Å were reported (Sigmon & Burns, 2010 ▶).

In the nitrato ligands, the N—O distances of the metal-coordinating oxygen atoms are, as expected, elongated [1.270 (3) to 1.287 (3) Å] compared to the N—O distances of the terminal oxygen atoms [1.210 (3) to 1.212 (3) Å]. Similar N—O distances were reported for the nitrate anions in Th(NO_3_)_4_(H_2_O)_4_ (Charpin *et al.*, 1987 ▶), Th(NO_3_)_4_(H_2_O)_5_ (Ueki *et al.*, 1966 ▶; Taylor *et al.*, 1966 ▶) and K_2_[Th(NO_3_)_6_] (Sigmon & Burns, 2010 ▶).

The *An*—O (*An* = Th) and N—O distances in the title compound are also comparable to the respective distances reported for the uranyl nitrate Rb(UO_2_)(NO_3_)_3_ (Zalkin *et al.*, 1989 ▶), with 2.474 (3) Å for *An*—O (*An* = U), 1.205 (6) Å for terminal N—O, and 1.268 (4) Å for the metal-coordinating oxygen atoms. The crystal chemistry of *M*[UO_2_(NO_3_)_3_] (*M* = K, Rb, and Cs) compounds, with *M* = K (Jouffret *et al.*, 2011 ▶; Krivovichev & Burns, 2004 ▶), Rb (Barclay *et al.*, 1965 ▶; Zalkin *et al.*, 1989 ▶) and Cs (Malcic & Ljubica, 1961 ▶), was discussed comparatively by Krivovichev & Burns (2004 ▶).

The caesium cation is surrounded by eleven NO_3_^−^-anions, one of which is chelating, leading to an overall coordination number of 12. The Cs—O distances of the chelating O-atoms range from 3.150 (2) to 3.436 (3) Å, whereas the other ten Cs—O distances are between 3.090 (2) and 3.552 (2) Å.

**Figure 2:**
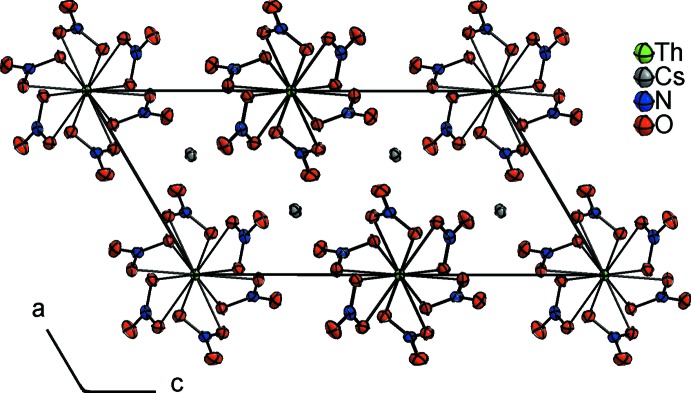
Unit cell of Cs_2_[Th(NO_3_)_6_] viewed along [010]. Displacement ellipsoids are shown at the 70% probability level.

The crystal structure of Cs_2_[Th(NO_3_)_6_] can be derived from a dense packing if the CsO_12_ and ThO_12_ units are idealized as spheres. The CsO_12_ units form a distorted hexa­gonal close-packed arrangement with the ThO_12_ units situated in half of the octa­hedral sites. The unit cell of Cs_2_[Th(NO_3_)_6_] is shown in Fig. 2 ▶, pointing out the pseudo-hexa­gonal arrangement.

The structure of the title compound is assumed to be isotypic with that of Rb_2_[Th(NO_3_)_6_] (Walker *et al.*, 1956 ▶), although atom positions have not been reported for the Rb compound so far. However, the unit cells are similar and the space group types are identical.

## Synthesis and crystallization   

0.1 g (0.18 mmol, 1 eq) Th(NO_3_)_4_·5H_2_O and 70 mg (0.36 mmol, 2 eq) CsNO_3_ were placed in a reaction flask and 100 ml water were added. The turbid solution was stirred and 1 ml of HNO_3_ conc. was additionally added, which led to a clear solution. The mixture was heated to 333 K and evaporated at 22 mbar in a rotary evaporator leading to a colourless powder. After dissolving the colourless solid in as little water as possible, the solution was allowed to evaporate at room temperature for one month. Single crystals of the title compound were obtained in an almost qu­anti­tative yield.

## Refinement   

Crystal data, data collection and structure refinement details are summarized in Table 1 ▶. The highest remaining electron density was found in Wyckoff position 2*a*. Inclusion of this density in the refinement led to unreasonable models. In the final model, this density was therefore not further considered.

## Supplementary Material

Crystal structure: contains datablock(s) global, I. DOI: 10.1107/S1600536814015876/wm5033sup1.cif

Structure factors: contains datablock(s) I. DOI: 10.1107/S1600536814015876/wm5033Isup2.hkl

CCDC reference: 1012751

Additional supporting information:  crystallographic information; 3D view; checkCIF report

## Figures and Tables

**Table 1:** Experimental details

Crystal data	
Chemical formula	Cs_2_[Th(NO_3_)_6_]
*M*_r_	869.92
Crystal system, space group	Monoclinic, *P*2_1_/*c*
Temperature (K)	123
*a*, *b*, *c* (Å)	8.1259 (14), 7.1873 (12), 15.583 (3)
β (°)	120.631 (10)
*V* (Å^3^)	783.1 (2)
*Z*	2
Radiation type	Mo *K*α
μ (mm^−1^)	14.22
Crystal size (mm)	0.09 × 0.07 × 0.06

Data collection	
Diffractometer	Bruker Kappa APEXII
Absorption correction	Multi-scan (*SADABS*; Bruker, 2008 ▶)
*T*_min_, *T*_max_	0.374, 0.498
No. of measured, independent and observed [*I* > 2σ(*I*)] reflections	31379, 3684, 2913
*R*_int_	0.043
(sin θ/λ)_max_ (Å^−1^)	0.831

Refinement	
*R*[*F* ^2^ > 2σ(*F* ^2^)], *wR*(*F* ^2^), *S*	0.023, 0.047, 1.04
No. of reflections	3684
No. of parameters	124
Δρ_max_, Δρ_min_ (e Å^−3^)	2.14, −1.35

Computer programs: *APEX2* and *SAINT* (Bruker, 2008 ▶), *SHELXS97* and *SHELXL97* (Sheldrick, 2008 ▶), *SHELXLE* (Hübschle *et al.*, 2011 ▶), *DIAMOND* (Brandenburg, 2012 ▶) and *publCIF* (Westrip, 2010 ▶).

## supplementary crystallographic information

### Crystal data

**Table:**

Cs_2_[Th(NO_3_)_6_]	*F*(000) = 772
*M**_r_* = 869.92	*D*_x_ = 3.689 Mg m^−^^3^
Monoclinic, *P*2_1_/*c*	Mo *K*α radiation, λ = 0.71073 Å
Hall symbol: -P 2ybc	Cell parameters from 9806 reflections
*a* = 8.1259 (14) Å	θ = 2.9–36.0°
*b* = 7.1873 (12) Å	µ = 14.22 mm^−^^1^
*c* = 15.583 (3) Å	*T* = 123 K
β = 120.631 (10)°	Block, colourless
*V* = 783.1 (2) Å^3^	0.09 × 0.07 × 0.06 mm
*Z* = 2	

### Data collection

**Table:**

Bruker Kappa APEXII diffractometer	3684 independent reflections
Radiation source: fine-focus sealed tube	2913 reflections with *I* > 2σ(*I*)
Graphite monochromator	*R*_int_ = 0.043
Detector resolution: 16 pixels mm^-1^	θ_max_ = 36.2°, θ_min_ = 2.9°
φ and ω scans	*h* = −13→13
Absorption correction: multi-scan (*SADABS*; Bruker, 2008)	*k* = −10→11
*T*_min_ = 0.374, *T*_max_ = 0.498	*l* = −25→25
31379 measured reflections	

### Refinement

**Table:**

Refinement on *F*^2^	0 constraints
Least-squares matrix: full	Primary atom site location: structure-invariant direct methods
*R*[*F*^2^ > 2σ(*F*^2^)] = 0.023	Secondary atom site location: difference Fourier map
*wR*(*F*^2^) = 0.047	*w* = 1/[σ^2^(*F*_o_^2^) + (0.0213*P*)^2^ + 0.6529*P*] where *P* = (*F*_o_^2^ + 2*F*_c_^2^)/3
*S* = 1.04	(Δ/σ)_max_ = 0.001
3684 reflections	Δρ_max_ = 2.14 e Å^−^^3^
124 parameters	Δρ_min_ = −1.35 e Å^−^^3^
0 restraints	

### Special details

**Table:**

Geometry. All e.s.d.'s (except the e.s.d. in the dihedral angle between two l.s. planes) are estimated using the full covariance matrix. The cell e.s.d.'s are taken into account individually in the estimation of e.s.d.'s in distances, angles and torsion angles; correlations between e.s.d.'s in cell parameters are only used when they are defined by crystal symmetry. An approximate (isotropic) treatment of cell e.s.d.'s is used for estimating e.s.d.'s involving l.s. planes.
Refinement. Refinement of *F*^2^ against all reflections. The weighted *R*-factor *wR* and goodness of fit *S* are based on *F*^2^, conventional *R*-factors *R* are based on *F*, with *F* set to zero for negative *F*^2^. The threshold expression of *F*^2^ > σ(*F*^2^) is used only for calculating *R*-factors(gt) *etc*. and is not relevant to the choice of reflections for refinement. *R*-factors based on *F*^2^ are statistically about twice as large as those based on *F*, and *R*-factors based on all data will be even larger.

### Fractional atomic coordinates and isotropic or equivalent isotropic displacement parameters (Å^2^)

**Table:**

	*x*	*y*	*z*	*U*_iso_*/*U*_eq_	
Th1	0.0000	0.5000	0.0000	0.00841 (3)	
Cs1	0.65173 (2)	0.75443 (3)	0.162303 (12)	0.01352 (4)	
N1	0.2073 (4)	0.7544 (4)	0.17622 (17)	0.0131 (4)	
O1	0.2995 (3)	0.8680 (4)	0.24064 (17)	0.0209 (5)	
O2	0.2851 (3)	0.6068 (3)	0.16636 (15)	0.0138 (4)	
O3	0.0296 (3)	0.7724 (3)	0.11416 (15)	0.0151 (4)	
N2	0.3383 (3)	0.2500 (4)	0.06501 (18)	0.0132 (4)	
O4	0.4570 (3)	0.1358 (4)	0.07556 (18)	0.0216 (5)	
O5	0.3172 (3)	0.4028 (3)	0.01760 (16)	0.0152 (4)	
O6	0.2249 (3)	0.2274 (3)	0.09779 (16)	0.0151 (4)	
N3	−0.1193 (3)	0.2394 (4)	0.10851 (18)	0.0119 (4)	
O7	−0.1394 (3)	0.1225 (3)	0.15830 (16)	0.0174 (4)	
O8	−0.1830 (3)	0.2212 (3)	0.01536 (15)	0.0146 (4)	
O9	−0.0265 (3)	0.3910 (3)	0.14759 (15)	0.0139 (4)	

### Atomic displacement parameters (Å^2^)

**Table:**

	*U*^11^	*U*^22^	*U*^33^	*U*^12^	*U*^13^	*U*^23^
Th1	0.00943 (5)	0.00874 (7)	0.00772 (5)	0.00017 (5)	0.00486 (4)	−0.00012 (5)
Cs1	0.01532 (8)	0.01297 (9)	0.01191 (7)	−0.00162 (6)	0.00667 (6)	0.00070 (6)
N1	0.0167 (10)	0.0129 (12)	0.0101 (9)	−0.0014 (9)	0.0071 (8)	−0.0003 (8)
O1	0.0273 (12)	0.0148 (12)	0.0158 (10)	−0.0061 (9)	0.0076 (9)	−0.0058 (8)
O2	0.0136 (9)	0.0135 (11)	0.0143 (9)	0.0002 (7)	0.0070 (8)	−0.0007 (7)
O3	0.0145 (9)	0.0172 (12)	0.0119 (9)	0.0006 (8)	0.0055 (7)	−0.0025 (8)
N2	0.0119 (10)	0.0151 (13)	0.0116 (9)	0.0032 (9)	0.0051 (8)	−0.0006 (9)
O4	0.0168 (10)	0.0203 (13)	0.0247 (11)	0.0088 (9)	0.0086 (9)	−0.0010 (9)
O5	0.0156 (9)	0.0164 (12)	0.0151 (9)	0.0021 (8)	0.0089 (8)	0.0023 (8)
O6	0.0157 (9)	0.0154 (12)	0.0165 (9)	0.0014 (7)	0.0098 (8)	0.0021 (8)
N3	0.0111 (9)	0.0123 (12)	0.0130 (10)	0.0023 (8)	0.0066 (8)	0.0032 (8)
O7	0.0226 (11)	0.0143 (12)	0.0200 (10)	0.0010 (8)	0.0142 (9)	0.0063 (8)
O8	0.0159 (9)	0.0177 (12)	0.0101 (8)	−0.0033 (8)	0.0065 (7)	−0.0021 (7)
O9	0.0177 (9)	0.0143 (11)	0.0116 (9)	−0.0028 (8)	0.0088 (8)	−0.0015 (7)

### Geometric parameters (Å, º)

**Table:**

Th1—O9	2.541 (2)	N1—O1	1.212 (3)
Th1—O9^i^	2.541 (2)	N1—O3	1.270 (3)
Th1—O5	2.547 (2)	N1—O2	1.283 (3)
Th1—O5^i^	2.547 (2)	O1—Cs1^iv^	3.090 (2)
Th1—O2	2.561 (2)	O2—Cs1^ii^	3.523 (2)
Th1—O2^i^	2.561 (2)	O3—Cs1^viii^	3.515 (2)
Th1—O3	2.573 (2)	N2—O4	1.212 (3)
Th1—O3^i^	2.573 (2)	N2—O6	1.271 (3)
Th1—O8^i^	2.578 (2)	N2—O5	1.285 (3)
Th1—O8	2.578 (2)	N2—Cs1^v^	3.584 (2)
Th1—O6^i^	2.581 (2)	O4—Cs1^ix^	3.109 (3)
Th1—O6	2.581 (2)	O4—Cs1^v^	3.436 (3)
Cs1—O1^ii^	3.090 (2)	O5—Cs1^v^	3.150 (2)
Cs1—O4^iii^	3.109 (3)	O6—Cs1^ii^	3.347 (2)
Cs1—O9^iv^	3.134 (2)	N3—O7	1.210 (3)
Cs1—O5^v^	3.150 (2)	N3—O8	1.275 (3)
Cs1—O7^vi^	3.161 (2)	N3—O9	1.287 (3)
Cs1—O2	3.194 (2)	N3—Cs1^ii^	3.657 (2)
Cs1—O6^iv^	3.347 (2)	O7—Cs1^x^	3.161 (2)
Cs1—O8^i^	3.385 (2)	O7—Cs1^ii^	3.624 (2)
Cs1—O4^v^	3.436 (3)	O8—Cs1^i^	3.385 (2)
Cs1—O3^vii^	3.515 (2)	O9—Cs1^ii^	3.134 (2)
Cs1—O2^iv^	3.523 (2)	O9—Cs1^viii^	3.785 (2)
Cs1—O5	3.552 (2)		
			
O9—Th1—O9^i^	180.0	O2—Cs1—O4^v^	111.59 (5)
O9—Th1—O5	111.46 (7)	O6^iv^—Cs1—O4^v^	169.89 (6)
O9^i^—Th1—O5	68.54 (7)	O8^i^—Cs1—O4^v^	62.74 (5)
O9—Th1—O5^i^	68.54 (7)	O1^ii^—Cs1—O3^vii^	103.26 (6)
O9^i^—Th1—O5^i^	111.46 (7)	O4^iii^—Cs1—O3^vii^	100.43 (6)
O5—Th1—O5^i^	180.0	O9^iv^—Cs1—O3^vii^	69.90 (5)
O9—Th1—O2	68.05 (7)	O5^v^—Cs1—O3^vii^	49.34 (5)
O9^i^—Th1—O2	111.95 (7)	O7^vi^—Cs1—O3^vii^	55.17 (6)
O5—Th1—O2	68.25 (7)	O2—Cs1—O3^vii^	160.35 (5)
O5^i^—Th1—O2	111.75 (7)	O6^iv^—Cs1—O3^vii^	116.33 (5)
O9—Th1—O2^i^	111.95 (7)	O8^i^—Cs1—O3^vii^	124.39 (5)
O9^i^—Th1—O2^i^	68.05 (7)	O4^v^—Cs1—O3^vii^	62.21 (5)
O5—Th1—O2^i^	111.75 (7)	O1^ii^—Cs1—O2^iv^	110.03 (6)
O5^i^—Th1—O2^i^	68.25 (7)	O4^iii^—Cs1—O2^iv^	62.67 (6)
O2—Th1—O2^i^	180.0	O9^iv^—Cs1—O2^iv^	50.38 (5)
O9—Th1—O3	68.29 (7)	O5^v^—Cs1—O2^iv^	152.33 (5)
O9^i^—Th1—O3	111.72 (7)	O7^vi^—Cs1—O2^iv^	62.66 (5)
O5—Th1—O3	113.67 (7)	O2—Cs1—O2^iv^	90.06 (4)
O5^i^—Th1—O3	66.33 (7)	O6^iv^—Cs1—O2^iv^	49.46 (5)
O2—Th1—O3	49.78 (7)	O8^i^—Cs1—O2^iv^	104.56 (5)
O2^i^—Th1—O3	130.22 (7)	O4^v^—Cs1—O2^iv^	120.70 (6)
O9—Th1—O3^i^	111.72 (7)	O3^vii^—Cs1—O2^iv^	109.23 (5)
O9^i^—Th1—O3^i^	68.28 (7)	O1^ii^—Cs1—O5	62.42 (6)
O5—Th1—O3^i^	66.33 (7)	O4^iii^—Cs1—O5	107.22 (6)
O5^i^—Th1—O3^i^	113.67 (7)	O9^iv^—Cs1—O5	152.10 (5)
O2—Th1—O3^i^	130.22 (7)	O5^v^—Cs1—O5	64.03 (6)
O2^i^—Th1—O3^i^	49.78 (7)	O7^vi^—Cs1—O5	145.89 (5)
O3—Th1—O3^i^	180.0	O2—Cs1—O5	49.93 (5)
O9—Th1—O8^i^	130.00 (7)	O6^iv^—Cs1—O5	111.39 (5)
O9^i^—Th1—O8^i^	50.00 (7)	O8^i^—Cs1—O5	48.65 (5)
O5—Th1—O8^i^	67.87 (7)	O4^v^—Cs1—O5	77.61 (6)
O5^i^—Th1—O8^i^	112.13 (7)	O3^vii^—Cs1—O5	111.10 (5)
O2—Th1—O8^i^	66.04 (7)	O2^iv^—Cs1—O5	139.61 (5)
O2^i^—Th1—O8^i^	113.96 (7)	O1—N1—O3	123.0 (3)
O3—Th1—O8^i^	67.52 (7)	O1—N1—O2	121.4 (3)
O3^i^—Th1—O8^i^	112.48 (7)	O3—N1—O2	115.6 (2)
O9—Th1—O8	50.00 (7)	O1—N1—Th1	171.90 (19)
O9^i^—Th1—O8	130.00 (7)	O3—N1—Th1	58.37 (14)
O5—Th1—O8	112.13 (7)	O2—N1—Th1	57.86 (13)
O5^i^—Th1—O8	67.87 (7)	O1—N1—Cs1	80.78 (16)
O2—Th1—O8	113.96 (7)	O3—N1—Cs1	135.65 (16)
O2^i^—Th1—O8	66.04 (7)	O2—N1—Cs1	56.08 (13)
O3—Th1—O8	112.48 (7)	Th1—N1—Cs1	93.18 (6)
O3^i^—Th1—O8	67.52 (7)	N1—O1—Cs1^iv^	151.9 (2)
O8^i^—Th1—O8	180.0	N1—O1—Cs1	80.51 (16)
O9—Th1—O6^i^	114.04 (7)	Cs1^iv^—O1—Cs1	115.37 (7)
O9^i^—Th1—O6^i^	65.96 (7)	N1—O2—Th1	97.02 (15)
O5—Th1—O6^i^	130.17 (7)	N1—O2—Cs1	104.44 (16)
O5^i^—Th1—O6^i^	49.83 (7)	Th1—O2—Cs1	116.83 (7)
O2—Th1—O6^i^	111.92 (7)	N1—O2—Cs1^ii^	113.20 (15)
O2^i^—Th1—O6^i^	68.08 (7)	Th1—O2—Cs1^ii^	105.02 (7)
O3—Th1—O6^i^	67.62 (7)	Cs1—O2—Cs1^ii^	118.46 (6)
O3^i^—Th1—O6^i^	112.38 (7)	N1—O3—Th1	96.77 (16)
O8^i^—Th1—O6^i^	67.90 (7)	N1—O3—Cs1^viii^	127.70 (16)
O8—Th1—O6^i^	112.10 (7)	Th1—O3—Cs1^viii^	109.60 (7)
O9—Th1—O6	65.96 (7)	O4—N2—O6	123.3 (3)
O9^i^—Th1—O6	114.04 (7)	O4—N2—O5	121.3 (2)
O5—Th1—O6	49.83 (7)	O6—N2—O5	115.4 (2)
O5^i^—Th1—O6	130.17 (7)	O4—N2—Th1	169.0 (2)
O2—Th1—O6	68.08 (7)	O6—N2—Th1	58.96 (13)
O2^i^—Th1—O6	111.92 (7)	O5—N2—Th1	57.49 (13)
O3—Th1—O6	112.38 (7)	O4—N2—Cs1^v^	73.20 (16)
O3^i^—Th1—O6	67.62 (7)	O6—N2—Cs1^v^	141.44 (17)
O8^i^—Th1—O6	112.11 (7)	O5—N2—Cs1^v^	60.20 (13)
O8—Th1—O6	67.90 (7)	Th1—N2—Cs1^v^	98.63 (7)
O6^i^—Th1—O6	180.0	N2—O4—Cs1^ix^	148.2 (2)
O1^ii^—Cs1—O4^iii^	156.25 (6)	N2—O4—Cs1^v^	87.06 (17)
O1^ii^—Cs1—O9^iv^	89.90 (6)	Cs1^ix^—O4—Cs1^v^	117.75 (7)
O4^iii^—Cs1—O9^iv^	99.74 (6)	N2—O5—Th1	97.33 (15)
O1^ii^—Cs1—O5^v^	93.71 (6)	N2—O5—Cs1^v^	99.06 (16)
O4^iii^—Cs1—O5^v^	100.55 (6)	Th1—O5—Cs1^v^	122.56 (8)
O9^iv^—Cs1—O5^v^	118.27 (5)	N2—O5—Cs1	114.09 (16)
O1^ii^—Cs1—O7^vi^	145.66 (6)	Th1—O5—Cs1	106.16 (7)
O4^iii^—Cs1—O7^vi^	54.04 (6)	Cs1^v^—O5—Cs1	115.97 (6)
O9^iv^—Cs1—O7^vi^	58.91 (6)	N2—O6—Th1	96.08 (16)
O5^v^—Cs1—O7^vi^	89.74 (6)	N2—O6—Cs1^ii^	125.14 (16)
O1^ii^—Cs1—O2	65.17 (6)	Th1—O6—Cs1^ii^	109.54 (7)
O4^iii^—Cs1—O2	91.62 (6)	O7—N3—O8	123.3 (3)
O9^iv^—Cs1—O2	123.54 (5)	O7—N3—O9	121.5 (2)
O5^v^—Cs1—O2	113.40 (5)	O8—N3—O9	115.2 (2)
O7^vi^—Cs1—O2	142.57 (6)	O7—N3—Th1	169.82 (18)
O1^ii^—Cs1—O6^iv^	60.69 (6)	O8—N3—Th1	58.82 (13)
O4^iii^—Cs1—O6^iv^	109.47 (6)	O9—N3—Th1	57.20 (12)
O9^iv^—Cs1—O6^iv^	50.84 (5)	O7—N3—Cs1^ii^	78.86 (16)
O5^v^—Cs1—O6^iv^	149.23 (6)	O8—N3—Cs1^ii^	137.03 (15)
O7^vi^—Cs1—O6^iv^	102.26 (6)	O9—N3—Cs1^ii^	56.39 (13)
O2—Cs1—O6^iv^	73.15 (5)	Th1—N3—Cs1^ii^	93.37 (6)
O1^ii^—Cs1—O8^i^	104.90 (6)	N3—O7—Cs1^x^	147.48 (19)
O4^iii^—Cs1—O8^i^	59.16 (6)	N3—O7—Cs1^ii^	82.01 (16)
O9^iv^—Cs1—O8^i^	154.64 (5)	Cs1^x^—O7—Cs1^ii^	126.22 (7)
O5^v^—Cs1—O8^i^	81.81 (5)	N3—O8—Th1	96.14 (16)
O7^vi^—Cs1—O8^i^	109.40 (6)	N3—O8—Cs1^i^	124.10 (15)
O2—Cs1—O8^i^	50.29 (5)	Th1—O8—Cs1^i^	110.23 (7)
O6^iv^—Cs1—O8^i^	119.24 (5)	N3—O9—Th1	97.60 (15)
O1^ii^—Cs1—O4^v^	129.25 (6)	N3—O9—Cs1^ii^	103.60 (16)
O4^iii^—Cs1—O4^v^	62.25 (7)	Th1—O9—Cs1^ii^	117.54 (7)
O9^iv^—Cs1—O4^v^	122.67 (6)	N3—O9—Cs1^viii^	110.88 (15)
O5^v^—Cs1—O4^v^	38.31 (6)	Th1—O9—Cs1^viii^	102.81 (7)
O7^vi^—Cs1—O4^v^	68.42 (6)	Cs1^ii^—O9—Cs1^viii^	121.84 (6)

Symmetry codes: (i) −*x*, −*y*+1, −*z*; (ii) −*x*+1, *y*−1/2, −*z*+1/2; (iii) *x*, *y*+1, *z*; (iv) −*x*+1, *y*+1/2, −*z*+1/2; (v) −*x*+1, −*y*+1, −*z*; (vi) *x*+1, *y*+1, *z*; (vii) *x*+1, *y*, *z*; (viii) *x*−1, *y*, *z*; (ix) *x*, *y*−1, *z*; (x) *x*−1, *y*−1, *z*.
